# Appropriateness and Outcome of a Statin Deprescription Intervention in Hospitalized Frail Older Adults: A Retrospective Study

**DOI:** 10.3390/geriatrics11020050

**Published:** 2026-04-20

**Authors:** Giuseppe Castiglia, Nicola Veronese, Gianluca Gortan Cappellari, Erica Voinovich, Paolo De Colle, Michela Zanetti

**Affiliations:** 1 Geriatric Clinic, Maggiore University Hospital, 34125 Trieste, Italy; giuseppe.castiglia@asugi.sanita.fvg.it (G.C.);; 2 Faculty of Medicine, Unicamillus Saint Camillus International University of Health Sciences, 00131 Rome, Italy; 3 Primary Care Department, Unità Locale Socio-Sanitaria 3 “Serenissima”, 30122 Venice, Italy; 4 Department of Medical, Surgery and Health Sciences, University of Trieste, 34127 Trieste, Italy; 5 UCO Geriatria, Ospedale Maggiore, Piazza dell’Ospitale 1, 34129 Trieste, Italy

**Keywords:** statin, deprescription, polypharmacy, older adults, mortality

## Abstract

**Background/Objectives**: The appropriateness of statin treatment in frail older adults is controversial because of insufficient evidence on its efficacy. The aim of this study was to assess the appropriateness of statin prescription at admission and discharge from hospital and the association of deprescription with one-year mortality in a cohort of older patients. **Methods**: Monocentric retrospective observational study of older (≥65 year) adults admitted to a Geriatric Unit. Patients underwent comprehensive geriatric assessment and prevalence of statin prescription at admission and discharge was recorded. Appropriateness of prescription was determined using the Medication Appropriateness Index (MAI), multidimensional frailty using the Multidimensional Prognostic Index (MPI). Mortality at 12 months was recorded. **Results**: Among 528 consecutively admitted patients, 112 (mean age 83.6 ± 6.2 years) were treated with statins and were included in the study. In addition, 87.5% of patients showed at least one inappropriate criterion for statin prescription at admission and 91.7% at discharge. Deprescription occurred in 46.4% of patients at discharge, particularly in those who were older and in MPI high mortality risk class. Mean MAI did not differ between admission and discharge in the whole study cohort, but it decreased in patients at highest mortality risk (from 3.2 ± 4.0 to 2.0 ± 1.2). In multivariate analysis, compared with patients who continued statins after discharge, those who were deprescribed did not show increased one-year mortality risk. **Conclusions**: Inappropriate statin prescription is common at hospital admission in frail older patients and deprescription does not affect one-year residual survival. Therefore, rigorous assessment of mortality risk and medication appropriateness should be encouraged.

## 1. Introduction

The global landscape is undergoing a rapid demographic shift toward population ageing, with the number of individuals aged 60 and over projected to rise from 1 billion in 2020 to 1.4 billion by 2030 and doubling to 2.1 billion by 2050 [1].

A large body of evidence supports the benefits of statins throughout all ages of life, including older ages. In the community setting, statin therapy in adults ≥ 75 years old is associated with lower risk of mortality [2,3,4,5] while discontinuation in those on long-term treatment results in a higher rate of cardiovascular events both when prescribed for primary and for secondary prevention [6,7,8]. In addition, in community-dwelling older adults statin use is generally safe, as the risk of severe adverse events appears to be low [3,9].

Beyond their established lipid-lowering role, statins exert significant antithrombotic, profibrinolytic, and antiplatelet effects. These anticoagulant properties appear to function through mechanisms independent of cholesterol reduction, as the clinical benefit does not directly correlate with the magnitude of LDL decrease. While these pleiotropic effects are more pronounced in patients with hypercholesterolemia, they represent a distinct pathway for cardiovascular protection that complements traditional lipid management [10]. Indeed, beyond their cholesterol-lowering action, statins exert pleiotropic effects with potential benefits on several diseases and conditions, including the possibility to delay the aging process [11] and the neuroprotective effect for all types of dementia [12].

Despite this evidence, the advantages of using statins in special groups of older adults such as those very old and living with frailty is still uncertain [13,14,15]. The prevalence of frailty typically increases in selected settings, such as hospitals, where it can be found in up to 47% of inpatients [16] and with advancing age, affecting up to 25% of those aged ≥85 [17]. Frailty is associated with multiple comorbidities, polypharmacy, inappropriate prescriptions and reduced life expectancy [18] that should be considered when assessing the appropriateness of statin use. The estimated time to prevent one major cardiovascular event by statins is 16 weeks in secondary prevention [19] but is 2.5 years in primary prevention so only those with an expected longer life expectancy may experience a survival benefit [20].

In addition, the risk of adverse reactions due to drug–drug and drug–disease interactions particularly in those living severe frailty may reduce the advantages of statin therapy. For these reasons, current guidelines do not provide clear recommendations for statin inception and deprescription in this population, whereas individualized assessment of risks and benefits, including a systematic evaluation of frailty, is highly suggested [21]. Indeed, quantification of mortality risk systematically using validated prediction tools to assess life expectancy (and therefore survival benefit) and identification of potentially inappropriate prescriptions by applying standardized scores may contribute to enhance the appropriateness of statin treatment. Hospitalization offers a valuable opportunity to review and address the indications for treatment taking into account patient’s frailty, comorbidities, polypharmacy and goals of therapy [22].

In older patients, therapeutic decision-making must shift from a disease-centric model to a multidimensional approach, where frailty, functional status, and comorbidity burden serve as the primary determinants of prognosis. Assessing frailty identifies physiological vulnerability beyond chronological age, while functional status prioritizes the maintenance of independence over biochemical targets. Furthermore, evaluating the total comorbidity burden is essential to balance competitive risks and guide deprescribing strategies. Integrating these factors ensures person-centered care, aligning interventions with biological reserve and life expectancy to avoid mistreatment in vulnerable populations. To assess mortality risk, the Multidimensional Prognostic Index derived from the Comprehensive Geriatric Assessment (CGA) has shown good discrimination and accuracy in predicting mortality and adverse clinical outcomes in older hospitalized adults both in the short- and long-term [23]. The MPI score is calculated from eight items of CGA used routinely in our department: ADL (Activities of Daily Living), IADL (Instrumental Activities of Daily Living), SPMSQ (Short Portable Mental Status Questionnaire), MNA (Mini Nutritional Assessment), ESS (Exton Smith Scale), CIRS (Cumulative Illness Rating Scale), number of drugs, social status. In addition, the validity of MPI in assessing the appropriateness of statin treatment in community-dwelling older adults has been previously demonstrated [24]. On the other hand, the Medication Appropriateness Index, specifically developed for use in older adults [25] has been extensively validated and is considered a reliable instrument to assess the appropriateness of prescribing and to identify potentially inappropriate prescriptions [26]. The MAI score is based on several criteria for each prescribed medication which assess drug indication and effectiveness, correct dose and directions, drug–drug and drug–disease interactions, duplications, duration of treatment and cost. A summated score for each medication based on a weighting scheme is available to determine the appropriateness of drug prescription [27].

Given this background, the aims of the study were (1) to retrospectively assess the appropriateness of statin prescription at admission and discharge in hospitalized older adults categorized by mortality risk; (2) to identify factors associated with statin deprescription; (3) to investigate whether statin deprescription is independently associated with increased risk of one-year mortality.

## 2. Materials and Methods

### 2.1. Study Design and Population

This was a retrospective observational study conducted in the Geriatric Clinic of Maggiore University Hospital in Trieste, Italy. Patients aged ≥65 years admitted to the ward for an acute medical condition or for a relapse of a chronic condition between 1 January 2019 and 31 December 2019 were screened for inclusion in the study. Exclusion criteria were (a) age < 65 years; (b) lack of informed consent to use clinical data for research purposes; and (c) lack of statin therapy at admission. The study was conducted in accordance with the Declaration of Helsinki and was approved by the Ethics Committee of the University of Trieste (approval number 127, date of approval: 23 January 2023) as part of the “Prevalence and prognostic impact of frailty and depression in hospitalized older adults” non-registered research protocol.

### 2.2. Data Collection

Information about patients’ demographics and living conditions were recorded at admission in the study ward. Clinical and laboratory data were extrapolated from the hospital electronic archive and from patients’ medical records. Laboratory parameters included in the study were obtained at admission. All participants underwent, within 72 h of admission to the ward, a standardized CGA for multidimensional evaluation, considering functioning, nutritional and cognitive status, social conditions and medication review. Number of medications at admission and discharge as well as statin prescription/deprescription were obtained from the CGA and from medical records. Medication was defined according to the Anatomical Therapeutics Chemical Classification standardized system.

Multidimensional Prognostic Index, based on standardized CGA and considering eight domains was used to assess the risk of mortality. Functional status was evaluated by Activities of Daily Living [28] to define the level of functional ability in basic care personal activities and by Instrumental Activities of Daily Living (IADL) to assess the level of independence in daily living [29]. Cognitive Status was assessed using the Short Portable Mental Status Questionnaire (SPMSQ) [30] while risk of developing pressure sores was estimated through the Exton Smith Scale (ESS) [31]. Nutritional status was determined using the Mini Nutritional Assessment (MNA) [32], comorbidities by the Cumulative Illness Rating Scale (CIRS) [33]. Living conditions were also recorded. Based on conventional cut-off points derived from the literature for every single item, each domain was assigned a standard score depending on the degree of impairment as follows: 0 = no issues, 0.5 = minor issues and 1.0 = major issues. The resulting sum of the calculated scores from the eight domains was then divided by 8 to obtain a final MPI risk score ranging from 0 = no risk of mortality to 1 = highest risk of mortality. The MPI was then expressed either as a continuous value or as three grades of mortality risk: MPI-1 = low risk (≤0.33), MPI-2 moderate risk (0.34–0.66), and MPI-3 high risk (>0.66) [23]. The MPI on admission was used for this study.

Appropriateness of statin prescription was evaluated using MAI. The tool, validated for its reliability both in ambulatory and hospital settings [26], measures the level of appropriateness of prescribing for older patients integrating clinical and laboratory information. The MAI consists of ten components which assess indication medication effectiveness and dosage, directions for use and practicality of the directions, drug–drug and drug–disease interactions, duplication of therapy, duration of treatment and costs [26]. To evaluate the correctness of drug dosage we used the ASCVD (Atherosclerotic Cardiovascular Disease guidelines). For drug–drug interactions, we checked the drugs information leaflets and we used a tool named Inter-Check Web (https://intercheckweb.marionegri.it/ accessed on 1 September 2024). To assess drug–disease interactions, we checked the presence of acute hepatic disease or chronic kidney impairment. For each criterion, the prescribed medication can be rated as appropriate, marginally appropriate, or inappropriate. Each criterion is then assigned a weighted score in the range between 1 and 3, with a possible maximum total score of 18, with higher scores reflecting a higher level of inappropriateness. The number and percentage of patients with at least one criterion rated as inappropriate by MAI criterion were also recorded. Two independent raters (experienced consultant geriatricians) independently reviewed the data and applied the MAI criteria. To reduce the chances of discrepancies occurring during data analysis and to agree on the methodology each evaluated criterion was preliminary analyzed and discussed and a consensus was reached on the possible rating. Survival status 1 year after discharge, was recorded. Overall mortality was considered as a primary outcome of the study. Vital status at 1 year was determined by consulting the hospital’s electronic records which are linked to the national mortality registry.

### 2.3. Statistical Analysis

Data were checked for normality of distribution via Shapiro–Wilk test. Variables were reported as mean ± standard deviation (SD), median with interquartile range [IQR] or frequency with relative percentage as appropriate. Subgroup comparison was performed by independent sample t test or Mann–Whitney for normally or non-normally distributed variables, respectively. Variable changes among MPI-stratified groups were assessed by the trend linear regression analysis. Similarity for percentage expressed data was assessed by the chi-square test. Fisher’s correction was applied, where necessary. Associations between variables and statin deprescription were evaluated by point-biserial correlation or chi-square for continuous and ordinal or dichotomous independent variables, respectively. Hazard ratios for mortality, with relative 95% confidence intervals, were determined by Cox-regression with stepwise multiple adjustments. Hazard ratios for mortality were also tested after propensity score matching, after balancing covariates of deprescribed and non-deprescribed groups according to Rosenbaum and Rubin [34]. *p* values < 0.05 were considered significant. Analyses were conducted using SPSS v. 17 (SPSS, Inc., Chicago, IL, USA).

## 3. Results

**Figure 1:**
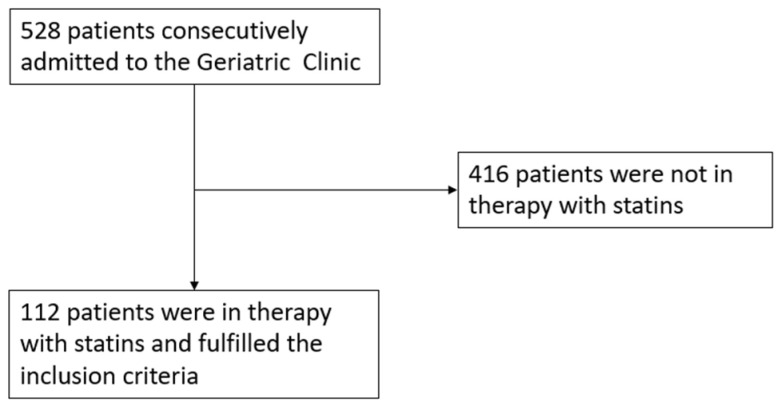
Patients included in the study.

Initially, 528 patients aged ≥65 years consecutively admitted to the Geriatric Department during the study period were screened. Of these, 112 (21.2%) were on statins and fulfilled the inclusion criteria (Figure 1). Therefore, the study population consisted of 112 patients, 54 men (48.2%) and 58 females (51.8%) with a mean age of 83.6 ± 6.2 years.

Table 1 shows the characteristics of the patients divided according to their MPI mortality risk class: 49 patients (43.8%) were in MPI-1 mild mortality risk class, 49 (43.8%) in MPI-2 moderate risk and 14 (12.5%) in MPI-3 severe risk of mortality class. Patients in moderate and severe risk classes were older and had lower BMI, were more functionally impaired, showed a higher risk of malnutrition and of developing pressure sores, had worse cognitive function and a higher number of comorbidities (all domains *p* for trend < 0.001). A lower (*p* < 0.001) percentage of patients in MPI-2 (moderate) and MPI-3 (severe) mortality risk class lived alone as compared to those in class 1 (mild). No significant differences were recorded among the three groups in the laboratory parameters. One-year mortality incidence rates were MPI-1 risk class: 22%; MPI-2 risk class: 45.8%; MPI-3 risk class: 71.4% (*p* for trend < 0.001).

Table 2 shows the indications for statin prescription at admission and discharge as well as the MAI score in the whole study cohort and according to mortality risk classes. On admission, 50% of patients were receiving statins for primary prevention and 50% for secondary prevention; a similar pattern was confirmed for patients in mild and moderate mortality risk classes while in severe mortality risk class there was a trend towards a higher (64.3%) percentage of patients treated for primary prevention. At discharge, the number of patients receiving statins decreased to 60 (53.6%). Deprescription occurred more frequently in those in higher mortality risk classes compared to those with lower risk (*p* = 0.017); in the severe mortality risk group, most of the patients who were deprescribed assumed statins for primary prevention. The average MAI score was similar on admission and discharge in the whole cohort; however, when considering MPI mortality risk classes, a decline (from 3.2 to 2) was observed selectively in high-risk class.

With regard to medication appropriateness, based on MAI, 87.5% of patients were receiving statins rated as inappropriate on at least one criterion at admission; this percentage was similar at discharge (Table 3). The most common identified criteria of inappropriateness at admission and at discharge were drug–drug interactions (46.4% and 48.3%) and incorrect dosage (44.6% and 48.3%, respectively).

When considering factors associated with statin deprescription, correlation analysis demonstrated significant (*p* ≤ 0.005) associations with older age, higher 1-year mortality risk, worse nutritional (as from albumin levels and MNA score) and functional status (ADL at discharge) and impaired renal function (Table 4).

Table 5 shows the association between statin deprescription and mortality over one year of follow-up. In unadjusted regression analysis, patients who were deprescribed did not present higher hazard ratio for mortality. Moreover, this finding was confirmed after age and MPI adjustment and by propensity score matching assessment, a method which allows for better evaluation of potential causal effects by reducing covariate’s impact (Supplementary Table S1). In addition, after stratifying for age and MPI mortality risk class, 1-year mortality was also not different among study groups.

## 4. Discussion

This is one of the first studies which investigated statin appropriateness and the effect of a statin deprescribing intervention during hospitalization on 1-year mortality based on validated tools to measure medication appropriateness and mortality risk among frail very old adults. Our study revealed that over 80% of patients assuming statins at admission were rated as inappropriate in at least 1 domain using standardized criteria and that in-hospital statin deprescription did not result in significantly increased 1-year mortality rate.

The cardiovascular benefits of statins in all age groups, including older adults have been extensively documented (2–5). In addition, the potential association of statin use with improved outcomes in conditions other than cardiovascular diseases like thrombosis and pulmonary embolism as suggested has also been demonstrated by recent evidence [35]. However, in very old adults living with frailty and approaching the end-of-life the appropriateness of statin therapy in primary and secondary prevention remains controversial, as the risks associated with adverse effects may outweigh the potential benefits. For this reason, available guidelines do not provide clear recommendations in this population and decisions should be individualized according to patient’s cardiovascular risk and functional status [21]. In our study, on admission statin prescription appropriateness and indications (primary vs. secondary prevention) were not significantly different among older patients at different 1-year mortality risk using the MPI, which is also validated for the assessment of multidimensional frailty [36]. However, when considering the single items rated as inappropriate at MAI, a high proportion of inappropriateness was recorded. A previous study by Adam et al. [37] found that, using a cardiovascular risk score to assess statin therapy appropriateness in hospitalized older patients with polypharmacy, almost 34% were at risk of inappropriate statin use with 18.2% potentially overusing statins, and 15.5% underusing them. Compared to these findings, the higher rate of older adults with at least one inappropriate criterion using MAI at admission in our study may be explained by the considerable prevalence of patients (>60%) using statins in primary prevention in MPI-3 mortality risk class; indeed, this subgroup showed the highest rate of deprescription at discharge. With this regard, a previous study reported that about 30% of older adults near the end-of-life continued drugs of questionable clinical benefit, including statins [38]. In addition, when considering the whole cohort independently of mortality risk, drug–drug interactions and dosage were the most represented criteria for statin inappropriate prescription both at admission and discharge. Similar findings have been reported by Tesfaye et al. in a cohort of hospitalized older adults with chronic kidney disease [39].

Hospitalization represents an important opportunity and a triage point to review and to deprescribe potentially inappropriate drugs [22] as medication review and deprescribing are associated with reduced hospital readmissions [40]. Indeed, once initiated, statin therapy needs to be periodically reassessed taking into account changes in the patient’s functional and medical status, possibly becoming no longer necessary or appropriate in the case of severe frailty or end-of-life. Indeed, recent studies have shown that in the end-of-life hospice care statins continue to be prescribed and are among the most common potentially inappropriate medications particularly in older adults [41,42]. In the absence of a formal deprescribing algorithm, application of a mortality risk score enhances the possibility of a better identification of older adults who may benefit from statin discontinuation; indeed, a previous study that considered indication (primary prevention) and age (≥75 years) as criteria for potentially statin deprescription during hospitalization showed that among those considered eligible, deprescription occurred in about 53% of patients; a similar rate of deprescription occurred in this study, with 70% of deprescription recorded among patients with high 1-year mortality risk. This finding is also in line with trends previously reported from UK, showing that statin deprescribing increases with frailty, age and primary prevention indication [43].

In addition to these parameters, impaired renal function was associated with statin discontinuation. Although the risk of renal toxicity during statin administration is generally low [44], polypharmacy in the context of frailty increases the risks of drug–drug interactions resulting in changes in statin pharmacokinetics. As a consequence, their plasma concentrations increase, being responsible for up to 50% of adverse events associated with statin use [45]. Indeed, polypharmacy, typical of frail multimorbid older patients and inappropriate prescribing show a bi-directional relationship which predispose to adverse drug reactions [46].

Although statins are generally safe and effective in all age groups, including older adults deprescription should be carefully undertaken because of the increased risk of cardiovascular events. Indeed, while deprescribing statins prescribed for primary prevention may be appropriate in selected individuals, particularly those with limited life expectancy, high frailty burden, or increased risk of adverse drug effects, given the delayed time-to-benefit of lipid-lowering therapy, greater caution should be warranted when considering deprescribing in the context of secondary prevention, where evidence supports continued benefit in reducing recurrent cardiovascular events, even in older populations. Thus, although patients’ stratification by mortality risk and by treatment appropriateness as in our study represents a valid opportunity for reasoned deprescribing other factors should be taken into account, including comorbidities, frailty, functional status and patient preferences.

The results of this study conducted in the hospital setting among frail very old, hospitalized patients demonstrate that statin deprescription is not associated with increased 1-year mortality; similar results have been reported by Kutner et al. [47] in a cohort of older adults approaching the end-of-life. In addition, a recent systematic review has confirmed that statin therapy may not be appropriate either in primary or secondary prevention in severely frail and completely dependent older adults as identified by a Clinical Frailty Scale score ≥ 7 [48]. In addition, discontinuing statin therapy in patients with reduced life expectancy may be associated with improved quality of life and use of fewer medications [47].

This study is only preliminary research and has some strengths and limitations. The main strength is the use of standardized tools to assess statin appropriateness and mortality risk derived from the comprehensive geriatric assessment. Despite the limited sample size, the inclusion of a homogenous population of hospitalized very old frail adults makes the results more compact from a methodological point of view. On the other hand, the single-institute nature of the study limits the generalizability of the findings. A significant limitation of this study is the one-year follow-up, a timeframe that may not be sufficient to fully capture the long-term clinical consequences of our intervention strategy on both overall mortality and the incidence of major cardiovascular events; because the protective benefits of statins tend to manifest cumulatively over time, more extensive monitoring would be necessary to precisely assess the safety and efficacy of statin deprescribing in this population. Another important limitation of our study is the small sample size which did not allow us to conduct a robust subgroup analysis of patients taking statins for primary or secondary prevention, as the clinical implications of deprescription may vary substantially between these groups. Besides potential residual confounding, even after propensity score matching, may limit causal inference. In addition, it should be noted that the MAI does not detect patients who were under prescribed; therefore, potential statin prescription omissions may have impacted on survival. It should be noted however that the rate of new statin users beyond 80 years old is as low as 2.4% per year and further declines with age (43). Finally, no information about effective statin suspension among those who were deprescribed following hospital discharge is available.

## 5. Conclusions

The importance of the appropriateness of statin prescription and deprescription in older adults remains an underrecognized issue, despite the growing awareness of the importance of statin treatment in both primary and secondary prevention across the lifespan. This is underscored by the limited number of studies addressing this issue in frail older population using standardized tools [46,47]. Indeed, the availability of validated instruments to predict mortality risk and treatment appropriateness offers clinicians an evidence-based support for evaluating the suitability of statin therapy in older adults for which evidence-based guidelines and recommendations are not available. Both the MAI and the MPI have demonstrated excellent reliability in assessing medication appropriateness and mortality risk and in older adults. Their integration in clinical practice can facilitate mortality risk stratification and individualized therapeutic decision-making. In patients with moderate to high 1-year mortality risk, deprescribing statins should be considered after a thorough evaluation of treatment appropriateness. In our sample, deprescription does not negatively impact 1-year survival; however, this is only a preliminary study subject to the limitations previously outlined. Further clinical and pharmacological research will be needed to provide individualized prognostic tools for assessing the risk-benefit ratio of prescribing and deprescribing statins in the elderly. Clinical questions to be addressed include, for example, the duration of therapy in relation to functional reserve, the estimation of the therapeutic window during polypharmacy and in the presence of geriatric syndromes and the possibility of monitoring the pleiotropic effects of statins over time.

## Figures and Tables

**Table 1:** Baseline characteristics of hospitalized older patients on statin treatment at admission and at discharge subdivided according to Multidimensional Prognostic Index (MPI) risk class. CGA: comprehensive geriatric assessment; ir %: incidence rate (number of events per patient group); CRP: C-reactive protein; eGFR: estimated glomerular filtration rate.

Variable	All	MPI-1 Mild Risk (%)	MPI-2 Moderate Risk (%)	MPI-3 Severe Risk (%)	*p* for Trend
Patients [*n*, (%)]	112	49 (43.8)	49 (43.8)	14 (12.5)	-
Age [y]	83.6 ± 6.2	81.3 ± 5	83.6 ± 6.7	86.9 ± 6.7	0.006
Gender, men [*n*, (%)]	54 (48.2)	26 (53.1)	22 (46.9)	6 (42.9)	0.664
BMI [kg/m^2^]	24.7 ± 4.7	24.5 ± 4.1	25.8 ± 5.1	21.6 ± 3.4	0.014
**CGA**					
Activities of Daily Living (ADL) [points]	4.1 ± 2.1	5.7 ± 0.9	4.6 ± 1.7	2.2 ± 0.9	<0.001
Instrumental Activities of Daily Living (IADL) [points]	4.8 ± 2.7	6.5 ± 1.7	4.3 ± 2.5	0.9 ± 1.2	<0.001
Short Portable Mental Status Questionnaire (SPMSQ) [points]	2.1 ± 2.6	0.9 ± 0.8	2.2 ± 2.4	5.9 ± 3.5	<0.001
Mini Nutritional Assessment (MNA) [points]	21.3 ± 6.0	23.7 ± 4.2	21.1 ± 6.2	13.8 ± 4.6	<0.001
Exton Smith Scale (ESS) [points]	15.6 ± 3.7	18.7 ± 1.6	16.9 ± 2.6	14.1 ± 2.1	<0.001
Cumulative Illness Scale (CIRS) [points]	5.2 ± 2.1	4.6 ± 1.9	5.5 ± 2.3	6.0 ± 1.4	0.017
Number of medications [*n*]	7.4 ± 2.8	6.7 ± 2.6	7.2 ± 2.9	7.9 ± 2.6	0.123
Living alone [*n*, (%)]	46 (41.1)	10 (22.4)	27 (55.1)	9 (64.3)	<0.001
Multidimensional Prognostic Index (MPI) [points]	0.40 ± 0.2	0.25 ± 0.06	0.47 ± 0.09	0.73 ± 0.06	<0.001
**Laboratory**					
CRP [mg/L]	54.1 ± 71.3	45.3 ± 73.6	58.2 ± 74.0	68.6 ± 51.9	0.512
Albumin [g/dL]	3.5 ± 0.4	3.5 ± 0.4	3.5 ± 0.4	3.3 ± 0.4	0.065
Creatinine [mg/dL]	1.2 ± 0.6	1.1 ± 0.5	1.2 ± 0.6	1.5 ± 0.9	0.128
eGFR [mL/min/m^2^]	65.3 ± 27.6	70.6 ± 29.2	62.4 ± 23.1	57.3 ± 34.1	0.174
Haemoglobin [g/dL]	11.7 ± 1.8	11.7 ± 2.0	11.7 ± 1.7	11.7 ± 1.8	0.999
**Outcomes**					
Length of hospital stay [d]	9.6 ± 6.5	8.2 ± 4.0	10.7 ± 7.8	11 ± 7.8	0.114
Mortality at 1-year ev [ir %]	43 (38.4)	11 (22)	22 (45.8)	10 (71.4)	<0.001

**Table 2:** Indications for statin prescription and MAI score at admission and discharge.

Statin Prescription	All	MPI-1 Mild Risk (%)	MPI-2 Moderate Risk (%)	MPI-3 Severe Risk (%)	*p* for Trend
Admission					
Primary prevention	56 (50)	23 (46.9)	24 (49.0)	9 (64.3)	0.440
Secondary Prevention	56 (50)	26 (53.1)	25 (51)	5 (35.7)	0.440
MAI per statin [points]	3.3 ± 2.8	3.2 ± 2.7	3.5 ± 2.7	3.2 ± 4.0	0.887
**Discharge**					
Statin suspended at discharge [*n*, (%)]	52 (46.4)	16 (32.7)	26 (53.1)	10 (71.4)	0.017
Suspended—Primary prevention [*n*, (%)]	31 (59.6)	11 (68.7)	13 (50.0)	7 (70.0)	0.127
Suspended—Secondary prevention [*n*, (%)]	21 (40.4)	5 (31.3)	13 (50.0)	3 (30.0)	0.115
MAI per statin [points]	3.07 ± 2.3	3.1 ± 2.4	3.2 ± 2.5	2 ± 1.2	0.646

MAI: medication appropriateness index.

**Table 3:** Distribution of statin inappropriate prescription according to the different criteria of MAI at admission and discharge.

Variable	Admission	Discharge	*p*
No. of Patients	112	60	-
At least one inappropriate rating for MAI [*n*, (%)]	98 (87.5)	55 (91.7)	0.406
Indication [*n*, (%)]	16 (14.3)	5 (8.3)	0.256
Effectiveness [*n*, (%)]	27 (24.1)	13 (21.7)	0.718
Dosage [*n*, (%)]	50 (44.6)	29 (48.3)	0.643
Correct directions [*n*, (%)]	0 (0)	0 (0)	-
Practical directions [*n*, (%)]	0 (0)	0 (0)	-
Drug–drug interaction [*n*, (%)]	52 (46.4)	29 (48.3)	0.812
Drug–disease interaction [*n*, (%)]	11 (9.8)	2 (3.3)	0.125
Duplication [*n*, (%)]	0 (0)	0 (0)	-
Duration [*n*, (%)]	24 (21.4)	11 (18.3)	0.631

MAI: medication appropriateness index.

**Table 4:** Association analysis of factors associated with statin deprescription in the study cohort.

Variable	Statin Deprescription	
	rho	*p*
Age	0.246	0.009
Gender, men	−0.074	0.437
BMI	−0.177	0.062
**CGA**		
Activities of Daily Living (ADL) admission	−0.154	0.106
Activities of Daily Living (ADL) discharge	−0.275	0.003
Instrumental Activities of Daily Living (IADL)	−0.173	0.067
Short Portable Mental Status Questionnaire (SPMSQ)	0.169	0.075
Mini Nutritional Assessment (MNA)	−0.196	0.038
Exton Smith Scale (ESS)	−0.213	0.024
Comorbidity index rating Scale (CIRS)	−0.007	0.944
Number of medications	−0.045	0.635
Living alone	0.169	0.075
Multidimensional Prognostic Index	0.262	0.005
MAI at admission	0.053	0.579
MAI at discharge	-	-
Statin at admission for primary prevention	0.179	0.059
**Laboratory**		
CRP [mg/L]	0.014	0.890
Albumin [g/dL]	−0.272	0.004
Creatinine [mg/dL]	0.168	0.076
eGFR [mL/min/m^2^]	−0.263	0.005
Blood glucose [mg/dL]	−0.092	0.336
Haemoglobin [g/dL]	−0.184	0.052

BMI: body mass index; CGA: comprehensive geriatric assessment; CRP: C-reactive protein; eGFR: estimated glomerular filtration rate.

**Table 5:** Raw and adjusted logistic regression and propensity score analyses on statin deprescription and 1-year mortality in the whole study cohort and by baseline MPI stratification.

	Deprescribed/Total	Adjusted Logistic Regression (Hazard Ratio) (95% Confidence Intervals) *p* Value	Propensity Score (Hazard Ratio) (95% Confidence Intervals) *p* Value
**Raw**			
All sample	52/112	1.665 (0.898–3.084) 0.105	0.949 (0.381–2.362) 0.910
MPI			
1 Mild risk	16/49	2.453 (0.684–8.794) 0.168	0.993 (0.102–9.690) 0.995
2–3 Moderate-severe risk	36/63	1.368 (0.659–2.837) 0.400	0.858 (0.311–2.370) 0.768
**Model 1—age, adjusted**			
All sample	52/112	1.496 (0.774–2.893) 0.231	0.938 (0.376–2.336) 0.890
MPI			
1 Mild risk	16/49	3.435 (0.843–13.990) 0.085	1.108 (0.089–13.810) 0.937
2–3 Moderate-severe risk	36/63	1.171 (0.545–2.517) 0.686	0.905 (0.325–2.514) 0.848
**Model 2—age, MPI adjusted**			
All sample	52/112	1.384 (0.705–2.715) 0.345	1.074 (0.424–2.717) 0.881
MPI			
1 Mild risk	16/49	3.872 (0.953–15.733) 0.058	2.104 (0.076–58.620) 0.661
2–3 Moderate-severe risk	36/63	1.155 (0.530–2.518) 0.717	1.240 (0.422–3.645) 0.696

## Data Availability

The datasets presented in this article are not readily available because the data are part of an ongoing study. Requests to access the datasets should be directed to Ethics Committee of the University of Trieste.

## Supplementary Materials

The following supporting information can be downloaded at https://www.mdpi.com/article/10.3390/geriatrics11020050/s1. Table S1: STROBE checklist v4.

## Abbreviations

The following abbreviations are used in this manuscript:

MAI	Medication Appropriateness Index
MPI	Multidimensional Prognostic Index
CGA	Comprehensive Geriatric Assessment
ADL	Activities of Daily Living
IADL	Instrumental Activities of Daily Living
SPMSQ	Short Portable Mental Status Questionnaire
MNA	Mini Nutritional Assessment
ESS	Exton Smith Scale
CIRS	Cumulative Illness Rating Scale
ASCVD	Atherosclerotic Cardiovascular Disease guidelines
